# Dual-release hydrocortisone improves body composition and the glucometabolic profile in patients with secondary adrenal insufficiency

**DOI:** 10.1007/s12020-024-03711-9

**Published:** 2024-02-12

**Authors:** Nanna Thurmann Jørgensen, Victor Brun Boesen, Stina Willemoes Borresen, Thea Christoffersen, Niklas Rye Jørgensen, Peter Plomgaard, Christina Christoffersen, Torquil Watt, Ulla Feldt-Rasmussen, Marianne Klose

**Affiliations:** 1 Department of Endocrinology and Metabolism, Copenhagen University Hospital, Rigshospitalet, Copenhagen, Denmark; 2 Department of Clinical Medicine, Faculty of Health and Medical Sciences, Copenhagen University, Copenhagen, Denmark; 3 Department of Clinical Pharmacology, Copenhagen University Hospital, Bispebjerg and Frederiksberg Hospital, Copenhagen, Denmark; 4 Department of Clinical Biochemistry, Copenhagen University Hospital, Rigshospitalet, Copenhagen, Denmark; 5 Department of Biomedical Sciences, Faculty of Health and Medical Sciences, Copenhagen University, Copenhagen, Denmark

**Keywords:** Dual-release hydrocortisone, Diurnal cortisol secretion, Body composition, Glucose metabolism, Adrenal insufficiency

## Abstract

**Purpose:**

Studies have suggested improved metabolic profiles in patients with adrenal insufficiency treated with dual-release hydrocortisone (DR-HC) compared with conventional hydrocortisone (C-HC). This study investigates the effect of DR-HC compared with C-HC treatment on five health variables: diurnal salivary cortisol/cortisone, body composition, bone health, glucose metabolism, lipids, and blood pressure.

**Methods:**

Prospective study of 27 participants (24 men) with secondary adrenal insufficiency with measurements during stable C-HC and 16 weeks after treatment switch to DR-HC.

**Outcomes:**

Diurnal salivary-cortisol/cortisone, body composition assessed by Dual-Energy X-ray absorptiometry scan, bone status indices (serum type I N-terminal procollagen [PINP], collagen type I cross-linked C-telopeptide [CTX], osteocalcin, receptor activator kappa-B [RANK] ligand, osteoprotegerin, and sclerostin), lipids, haemoglobin A_1c_ (HbA_1c_), and 24-hour blood pressure.

**Results:**

After the switch to DR-HC, the diurnal salivary-cortisol area under the curve (AUC) decreased non-significantly (mean difference: −55.9 nmol/L/day, *P* = 0.06). The salivary-cortisone-AUC was unchanged. Late-evening salivary-cortisol and cortisone were lower (−1.6 and −1.7 nmol/L, *P* = 0.002 and 0.004). Total and abdominal fat mass (−1.5 and −0.5 kg, *P* = 0.003 and 0.02), HbA_1c_ (−1.2 mmol/mol, *P* = 0.02), and osteocalcin decreased (−7.0 µg/L, *P* = 0.03) whereas sclerostin increased (+41.1 pg/mL, *P* = 0.0001). The remaining bone status indices, lipids, and blood pressure were unchanged.

**Conclusion:**

This study suggests that switching to DR-HC leads to lower late-evening cortisol/cortisone exposure and a more favourable metabolic profile and body composition. In contrast, decreased osteocalcin with increasing sclerostin might indicate a negative impact on bones.

**Clinical trial registration:**

EudraCT201400203932

## Introduction

Adrenal insufficiency is a life-threatening condition [1] that requires substitution therapy with glucocorticoids [2].

Still, despite treatment, adrenal insufficiency is characterised by higher morbidity and mortality compared with the background population [3–6]. One explanation for the increased morbidity and mortality of the patients could be that current glucocorticoid treatment regimens induce unphysiological cortisol exposure [7].

Orally administrated hydrocortisone is the conventionally used glucocorticoid replacement therapy (C-HC) [8]. A thrice-daily regimen of C-HC with the highest dosage in the morning most accurately mirrors the circadian, physiological cortisol secretion [9]. Still, treatment is far from optimal due to a half-life of 1.8 h [10] causing peaks and throughs both under and over-shooting the physiological cortisol secretion [11, 12].

In 2009, the once-daily dual-release formulation of hydrocortisone (DR-HC) was introduced which seemed to mimic the physiological cortisol secretion more precisely than C-HC [13]. Several studies have shown promising results of improved cardiovascular, immune, metabolic profiles, and quality of life when converting from C-HC to DR-HC even though not all studies found these improvements significant or clinically relevant [14–26]. We have previously reported a reduction in fatigue, measured by ecological momentary assessment, in 9 of the 27 included participants with secondary adrenal insufficiency [22].

In the present paper, we report the effect of DR-HC compared with C-HC across a spectrum of health variables, including diurnal salivary cortisol/cortisone, body composition, bone status indices, glucose metabolism, lipids, and blood pressure in the previously included participants with secondary adrenal insufficiency. We hypothesise that an improved circadian profile of cortisol during DR-HC treatment will improve these health variables, especially in the group of participants with reduced fatigue.

## Methods

### Participants

Participants were patients ≥18 years old with secondary adrenal insufficiency in stable treatment with C-HC twice or thrice daily (total daily dose of 10–40 mg) followed at the Department of Endocrinology and Metabolism, Rigshospitalet, Copenhagen University Hospital, Denmark. Exclusion criteria were pregnancy or breastfeeding, diagnosis of acromegaly, Morbus Cushing, diabetes mellitus or other major confounding diseases, known or expected hypersensitivity to any of the excipients, and poor previous compliance.

We attempted to include primarily men and subsequently women not treated with oestrogen to minimise the influence of oestrogen on hormone-binding proteins such as the corticosteroid-binding protein. Pituitary function tests were not part of the study design. Still, all participants were at least yearly evaluated for all pituitary axes as part of the routine evaluation in the outpatient clinic.

The study was approved by the Regional Scientific Ethical Committee in Copenhagen (ID H-1-2014-073) and was conducted according to the Declaration of Helsinki. All participants received written and verbal information about the study and gave informed consent before enrolment. The Good Clinical Practice unit at Copenhagen University Hospital, Rigshospitalet monitored the study.

### Study design and outcome measures

The PlenadrEMA study was a prospective investigator-initiated open-label switch trial conducted at the Department of Endocrinology and Metabolism, Rigshospitalet, Copenhagen University Hospital, Denmark. The primary outcome was fatigue measured by ecological momentary assessments evaluated at baseline on C-HC and repeated 16 weeks after a switch in treatment to DR-HC Plenadren® [22, 27]. In the present study, the secondary outcomes included in the trial were analysed (Table 1, white table-cells).

**Table 1:** Study visits and outcomes

Grey cells: Outcomes previously analysed and published [22].

White cells: Outcomes analysed in the present study. The outcomes were measured at baseline on treatment with conventional hydrocortisone (visit 2/week 0) and 16 weeks after the switch to dual-release hydrocortisone (visit 4/week 16)

*Lipids (cholesterol, high-density lipoprotein cholesterol [HDL], low-density lipoprotein cholesterol [LDL], and triglycerides), fasting blood glucose, haemoglobin A1c, and bone status indices (serum type I N-terminal procollagen [PINP], collagen type I cross-linked C-telopeptide [CTX], osteoprotegerin [OPG], receptor activator kappa-B [RANK] ligand, osteocalcin, and sclerostin)

The saliva samples were analysed for cortisol and cortisone concentrations and collected using the absorbent synthetic fibre roll in Salivette® Cortisol at 0700, 1200, and 2300 hours for three days. The participants were instructed to stay relaxed, remove any lipstick, and refrain from tooth brushing, smoking, chewing gum, drinking, or eating 30 minutes before collecting the saliva sample. Afterwards, they were instructed to store the samples at +5 °C and take their dose of C-HC or DR-HC.

The blood samples were collected between 0800 and 1200 h after an overnight fast and at least 12 h after the last hydrocortisone dosage. Blood was analysed for concentrations of lipids (cholesterol, high-density lipoprotein cholesterol [HDL], low-density lipoprotein cholesterol [LDL], and triglycerides), fasting blood glucose, haemoglobin A1c (HbA_1c_), and bone status indices (serum type I N-terminal procollagen [PINP], collagen type I cross-linked C-telopeptide [CTX], osteocalcin, receptor activator kappa-B [RANK] ligand, osteoprotegerin, and sclerostin).

The dual-energy X-ray absorptiometry (DXA) scan (model XP-26/XR-46; Norland Medical Systems, Fort Atkinson, WI) was performed as a whole-body scan with a separate assessment of total fat and lean mass. Regional assessments of abdominal fat and lean mass were also obtained. Furthermore, BMD was measured at the lumbar spine (L2 to L4) and femoral neck and only evaluated at baseline on C-HC.

The 24-hour blood pressure monitoring was performed using a Spacelabs Healthcare Ultralite™ 90217 A monitor with measurements at 20-minute intervals during daytime (fixed interval: 0700–2200 h) and 30-minute intervals during the night (fixed interval: 2200–0700 h). The threshold for 24-hour hypertension was a mean blood pressure ≥130/80 mmHg, for daytime hypertension a mean ≥ 135/85 mmHg, and night-time hypertension was defined as a mean blood pressure ≥ 120/70 mmHg. Non-dipping during night-time was defined as a reduction of <10% in nocturnal blood pressure [28, 29].

### Study intervention

DR-HC was compared with the usual C-HC replacement regime of the participants.

Doses of C-HC included variable combinations of 20 mg tablets (TAKEDA) and an unlicensed preparation of hydrocortisone 5 mg tablets produced by Glostrup Pharmacy A/S. The total daily dose was between 10 and 40 mg administered twice to thrice daily.

DR-HC was delivered by Shire in tablets containing 5 and 20 mg. The participants received a daily dose of DR-HC equal to their usual daily dose of C-HC, however, the dose could be altered according to the clinical response as per usual clinical care. Also, as DR-HC was only available in doses of 5 and 20 mg, participants receiving intermediate doses of C-HC were rounded up to the nearest 5 mg. The participants were instructed to take the DR-HC tablets once daily in a fasting state upon awakening (between 0600 and 0800 h).

Participants were instructed to take and register additional substitution of C-HC tablets according to national guidelines in case of intercurrent illness, stress, or moderate-high-intensity exercise. At the end of the study, the participants resumed their usual treatment with C-HC.

## Analytical methods

Salivary cortisol/cortisone concentrations were measured by liquid chromatography-mass spectrometry by use of ^13C-labelled^ intern standard for cortisol and cortisone using Waters Xevo TQ-S mass spectrometer (Waters, Milford, Massachusetts, USA). The coefficient of variation (CV) for cortisol and cortisone was 8%. Concentrations of fasting glucose and lipids were analysed by enzymatic methods (absorption photometry) performed on a Cobas 8000 (Roche Diagnostics GmBH, Switzerland). The local, long-term maximal total assay coefficient of variation (CV_max_) of glucose was 4% at concentrations of both 3.5 and 20.0 mmol/L. The CV_max_ of cholesterol was 5% at the concentrations 3 and 7 mmol/L, the CV_max_ of HDL was 4% at both 0.9 and 2.2 mmol/L, the CV_max_ of LDL was 4% at both 1.7 and 4.9 mmol/L, and the CV_max_ of triglycerides was 5% at concentrations of 1.0 and 2.2 mmol/L.

Concentrations of HbA_1c_ were measured by liquid chromatography-absorption photometry performed on a Tosoh G8 HPLC Analyser (Tosoh Bioscience, Inc., United States). The CV_max_ of HbA_1c_ was 2.8% at concentrations of both 34 and 80 mmol/mol.

Plasma PINP, CTX, and osteocalcin were measured by an automated chemiluminescence immunoassay (CLIA) performed on an IDS-iSYS (Immunodiagnostic Systems Holdings Ltd, United Kingdom). The CV_max_ of PINP, CTX, and osteocalcin were 8% at concentrations of both 30 and 100 µg/L, 210 and 2110 ng/L, and 10 and 40 ug/L, respectively. Serum RANKL was analysed in one batch using the Human Bone Metabolism RANKL Magnetic Bead Single Plex Kit (Milliplex ®, Merck KGaA, Darmstadt, Germany) with an intra-assay CV of 15%. Plasma OPG was measured in one batch by sandwich ELISA (Biomedica Immunoassays, Biomedica Medizinprodukte GmbH, Austria) with an intra-assay CV of 12%. Serum sclerostin was measured by automated CLIA performed on a LIAISON® sclerostin assay (DiaSorin, Italy) with an intra-assay CV of <5% [30].

## Statistical analyses

All statistical analyses were made using SAS Enterprise Guide 8.3 (SAS Institute Inc., Cary, NC, USA). Categorial data are presented as numbers, *N* (%) and continuous data are presented as mean [95% confidence interval (CI)] if normally distributed. The continuous variables RANKL, CTX, the night-to-day ratio of systolic blood pressure, fasting glucose, triglycerides, and salivary cortisol and cortisone followed a log-normal distribution and are presented as median (interquartile range) unless otherwise stated. The diurnal salivary cortisol and cortisone productions were evaluated by AUC using the trapezoid rule. The AUCs also followed a log-normal distribution.

As the study was designed as a switch study, the participants were their own controls. Hence, differences between data before and after switching to DR-HC were evaluated with paired *t*-tests for quantitative data and Chi^2^-tests or Fisher’s exact tests for categorical data. To evaluate differences between subgroups, unpaired *t*-tests were performed. A univariate regression analysis was performed to investigate the relationship between concentrations of bone status indices and glucocorticoid replacement dose per kg body weight. The level of significance was set to *P* < 0.05.

## Results

### Participant characteristics

We included 30 participants with secondary adrenal insufficiency and 27 completed the study. Reasons for discontinuation were drop-out (*N* = 2) and exclusion from analyses (*N* = 1). Causes for drop-out were worsening of known Mb. Meniere (*N* = 1) and dissatisfaction with the DR-HC treatment (*N* = 1). One participant was hospitalised for months following a syncope and diagnosed with dilated cardiomyopathy (*N* = 1). Furthermore, this participant was treated for several infections, discontinued treatment with DR-HC and was thus excluded from analyses.

The clinical demographics of the 27 participants are given in Table 2. During the study period, the dose of DR-HC was increased in four participants due to worsening of fatigue (three participants from 20 to 25 mg, one participant from 30 to 35 mg), and in one participant, the dose was decreased upon request from 35 to 30 mg. After the alterations, the median daily dose of DR-HC was increased compared with C-HC. No differences in the intake of supplemental C-HC were found between the two treatment periods. In one participant, the thyroid hormone substitution treatment was lowered during the study period, but in all other participants, supplemental treatment with pituitary hormones was stable.

**Table 2:** Participant characteristics and recorded adverse events

	Participants (*N* = 27)
Sex (male/female)	24/3^#^
Age, years	62 [38 to 73]
Years since diagnosis	10 [1 to 41]
Hydrocortisone replacement dose	
<20 mg	3 (11)
20 to 25 mg	22 (82)
>25 mg	10 (37)
Hydrocortisone dose	20 [10 to 35]
Dual-release hydrocortisone dose	25 [10 to 35]
Hydrocortisone treatment regimen	
Twice daily	8 (30)
Thrice daily	19 (70)
Additional hormone replacement	
Growth hormone	19 (70)
Testosterone	19 (70)
Oestrogen	0
Thyroid hormone	25 (93)
Antidiuretic hormone	5 (19)
>3 deficient axes	5 (19)
Bone health (*N* = 22)*	
BMD T-score	
Lumbar spine (L2−L4)	−0.6 [−1.3 to 4.4]
Femoral neck	−1.0 [−2.4 to 1.6]
Anti-osteoporotic treatment	
Calcium and vitamin D treatment	3 (14)
Other anti-osteoporotic treatment	0
Adverse events (*N* = 18/30)**	
Total adverse events	35
Serious adverse events^	4 (11)
Atrial fibrillation leading to electrical cardioversion	2 (50)^^
Diagnosis of dilated cardiomyopathy	1 (25)
Pre-existing pituitary tumour growth	1 (25)

Results are expressed as *N* (%) or median [range]

*BMD*, bone mass density

^#^The three women were post-menopausal

*5 male patients were not scanned due to body weight >100 kg (*N* = 3) and scan malfunction (*N* = 2)

**Adverse events were recorded by 18 participants of all participants (*N* = 30) who entered the study

^The serious adverse events were not considered related to the intervention drug

^^The same participant

All reported adverse events are previously described [24]

### Diurnal salivary cortisol and cortisone

**Fig. 1:**
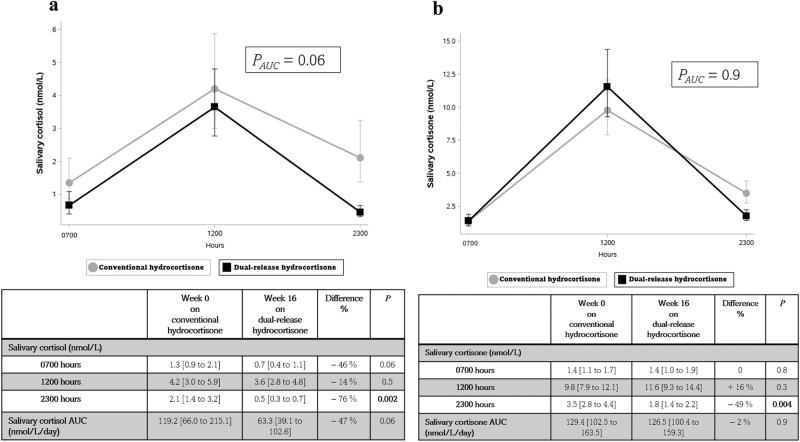
**a** Mean diurnal salivary cortisol at 0700, 1200, and 2300 h at week 0 on conventional hydrocortisone (grey circle) and week 16 on dual-release hydrocortisone (black square). Results are expressed as back-transformed mean [95% CI] and the percentage difference (%). Error bars represent 95% CI. Analyses were performed using paired *t*-tests *P*-values < 0.05 are presented in bold. **b** Mean diurnal salivary cortisone at 0700, 1200, and 2300 h at week 0 on conventional hydrocortisone (grey circle) and week 16 on dual-release hydrocortisone (black square). Results are expressed as back-transformed mean [95% CI] and the percentage difference (%). Error bars represent 95% CI. Analyses were performed using paired *t*-tests. *P*-values < 0.05 are presented in bold

The comparison of diurnal salivary cortisol and cortisone concentrations before and after 16 weeks of DR-HC is displayed in Fig. 1a, b.

After 16 weeks of DR-HC, the salivary cortisol AUC was lower with non-significantly lower concentrations at both 0700 and 1200 h but significantly lower concentrations at 2300 h.

The salivary cortisone AUC did not change. Salivary cortisone was unchanged at 0700 h and increased non-significantly at 1200 h but decreased significantly at 2300 h.

### Body composition

Body weight and BMI were unchanged after 16 weeks of DR-HC, whereas total and abdominal fat mass were significantly reduced compared with C-HC (Table 3). The total lean mass did not change.

**Table 3:** Body composition assessed by dual-energy X-ray absorptiometry (DXA) scan and glucose-metabolic outcomes on treatment with conventional hydrocortisone (week 0) and after 16 weeks on dual-release hydrocortisone treatment (week 16)

	Week 0 on conventional hydrocortisone	Week 16 on dual-release hydrocortisone	Difference	*P*
Body composition (*N* = 22)*				
Weight, *kg*	85.2 [79.0 to 91.5]	84.5 [78.3 to 90.8]	−0.7 [−1.7 to 0.3]	0.2
Body mass index, *kg/m*^*2*^	27.5 [25.8 to 29.1]	27.2 [25.6 to 28.9]	−0.3 [−0.6 to 0.1]	0.2
Total fat mass, *kg*	21.6 [18.6 to 24.5]	20.1 [17.7 to 22.5]	−1.5 [−2.3 to −0.6]	**0.003**
Total lean mass, *kg*	60.2 [55.2 to 65.1]	60.8 [56.0 to 65.5]	0.6 [−0.5 to 1.7]	0.3
Abdominal fat mass, *kg*	5.1 [4.4 to 5.9]	4.6 [4.1 to 5.3]	−0.5 [−0.8 to −0.1]	**0.02**
Glucose-metabolic outcomes				
Haemoglobin A1c, *mmol/mol*	35.5 [33.8 to 37.1]	34.3 [32.8 to 35.8]	−1.2 [−2.0 to −0.3]	**0.02**
Fasting glucose, *mmol/L*	5.0 (4.7 to 5.1)	5.0 (4.6 to 5.2)	0.0 [−0.4 to 0.2]	0.4
Total cholesterol, *mmol/L*	5.2 [4.9 to 5.6]	5.3 [4.9 to 5.7]	0.1 [−0.2 to 0.4]	0.4
HDL cholesterol, *mmol/L*	1.4 [1.3 to 1.6]	1.4 [1.2 to 1.5]	0.0 [−0.1 to 0.0]	0.3
LDL cholesterol, *mmol/L*	3.4 [3.0 to 3.7]	3.5 [3.2 to 3.8]	0.1 [0.0 to 0.4]	0.09
Triglycerides, *mmol/L*	1.5 (1.1 to 2.1)	1.6 (1.2 to 2.1)	0.1 [−0.5 to 0.4]	0.7

Blood samples were collected after an overnight fast and at least 12 h after the last hydrocortisone dosage. Results are expressed as mean [95% CI] or median (interquartile range). Analyses are performed using paired *t*-tests. *P*-values < 0.05 are presented in bold

*HDL* high-density lipoprotein, *LDL* low-density lipoprotein

*5 male participants were not scanned due to body weight >100 kg (*N* = 3) and scan malfunction (*N* = 2)

### Glucose-metabolic outcome

The results are seen in Table 3. HbA_1c_ significantly decreased after 16 weeks of DR-HC treatment compared with C-HC. No change was observed for fasting glucose and lipids after the switch in treatment.

### Blood pressure

The results of the 24-hour ambulatory blood pressure measurements before and after the 16 weeks of DR-HC treatment are shown in Table 4.

**Table 4:** 24-hour ambulatory blood pressure on treatment with conventional hydrocortisone (week 0) and after 16 weeks on dual-release hydrocortisone treatment (week 16)

24-hour ambulatory blood pressure (*N* = 23^)	Week 0 on conventional hydrocortisone	Week 16 on dual-release hydrocortisone	Difference	*P*
Mean blood pressure				
Systolic blood pressure, *mmHg*	124.7 [116.0 to 133.3]	122.3 [114.8 to 129.8]	−2.4 [−5.8 to 1.1]	0.2
Diastolic blood pressure, *mmHg*	77.0 [72.0 to 82.0]	75.6 [71.0 to 80.1]	−1.4 [−3.3 to 0.5]	0.1
Daytime (0700–2200 h)				
Systolic blood pressure, *mmHg*	128.2 [119.4 to 137.0]	125.7 [117.8 to 133.6]	−2.5 [−6.1 to 1.1]	0.2
Diastolic blood pressure, *mmHg*	80.1 [74.8 to 85.5]	78.7 [73.9 to 83.4]	−1.4 [−3.7 to 0.8]	0.2
Night-time (2200–0700 h)				
Systolic blood pressure, *mmHg*	116.2 [107.9 to 124.6]	114.9 [107.5 to 122.3]	−1.3 [−5.6 to 3.0]	0.6
Diastolic blood pressure, *mmHg*	69.1 [64.7 to 73.5]	69.2 [64.7 to 73.6]	0.1 [−2.3 to 2.5]	0.9
Night-to-day-ratio				
Systolic blood pressure	0.89 (0.9 to 1.0)	0.91 (0.9 to 1.0)	0.01 [−0.02 to 0.04]	0.5
Non-dipping	10 (43)	13 (57)	3 (14)	0.8
Hypertension subgroup (*N* = 7) ^^				
Mean blood pressure				
Systolic blood pressure, *mmHg*	151.3 [144.4 to 158.2]	143.4 [136.2 to 150.6]	−7.9 [−14.8 to −1.0]	**0.03**
Diastolic blood pressure, *mmHg*	89.4 [78.9 to 100.0]	84.7 [74.0 to 95.5]	− 4.7 [− 8.4 to −1.0]	**0.02**

Blood pressure was measured at 20-minute intervals during the daytime (0700–2200 h) and 30-minute intervals during the night (2200–0700 h). Non-dipping was defined as a night-to-day systolic blood pressure ratio >0.9. The threshold for 24-hour hypertension was a mean blood pressure ≥130/80 mmHg, for daytime hypertension a mean blood pressure ≥135/85 mmHg, and night-time hypertension was defined as a mean blood pressure ≥120/70 mmHg

Results are expressed as *N* (%), mean [95% CI] or median (interquartile range). Analyses are performed using paired *t*-tests and Chi^2^ test. *P*-values < 0.05 are presented in bold

^Four participants were not monitored (3 men) due to missing scans during the night-time (*N* = 3), and one participant declined

^^Seven participants had hypertension according to the definitions of the present study

No significant difference in any blood pressure measures was observed after 16 weeks of DR-HC treatment compared with baseline on C-HC.

A subgroup analysis of seven participants with hypertension (defined as a mean 24-hour blood pressure ≥130/80 mmHg) showed that the systolic and diastolic blood pressure decreased after the switch in treatment to DR-HC. Despite the decrease, they still met the criteria for hypertension. Only one of these participants was on antihypertensive treatment (ACE inhibitor) which was unchanged during the study period. The blood pressure of the participants without hypertension did not change (data not shown).

### Bone metabolism

Data on BMD and bone-related medical treatments are given in Table 2. The results of the bone status indices RANKL, OPG, PINP, osteocalcin, sclerostin, and CTX at baseline on C-HC and after 16 weeks of DR-HC are seen in Table 5. Osteocalcin was significantly lower, and sclerostin was significantly higher after 16 weeks of DR-HC. PINP, CTX, RANKL, and OPG were unchanged. A positive relationship was found between the change in sclerostin concentration and replacement dose per kg body weight (slope [95% CI]: 293.1 [3.0 to 583.2], *P* = 0.05) whereas no relationship was found between the change in concentrations of the remaining bone status indices and treatment dose per kg body weight (data not shown). In a subgroup analysis, no relationship was found between the bone status indices and the participants who registered intake of supplemental C-HC (*N* = 10) (data not shown).

**Table 5:** Bone status indices on treatment with conventional hydrocortisone (week 0) and after 16 weeks on dual-release hydrocortisone treatment (week 16)

	Week 0 on conventional hydrocortisone	Week 16 on dual-release hydrocortisone	Difference	*P*
PINP (µg/L)	70.8 [54.0 to 87.7]	57.1 [47.4 to 66.7]	− 13.8 [− 31.9 to 4.4]	0.13
CTX (ng/L)	264.1 (278.8 to 547.9)	310.3 (188.5 to 461.7)	− 60.9 [− 170.2 to 48.4]	0.26
OPG (pmol/L)	3.3 [2.8 to 3.8]	3.3 [2.9 to 3.8]	0.0 [− 0.3 to 0.4]	0.93
RANKL (pg/mL)	48.0 (33.0 to 167.0)	54.0 (35.0 to 186.0)	3.6 [− 2.3 to 9.4]	0.23
Osteocalcin (µg/L)	31.3 [14.8 to 41.5]	24.3 [19.8 to 28.8]	− 7.0 [− 13.4 to − 0.6]	**0.03**
Sclerostin (pg/mL)	443.3 [376.6 to 509.9]	484.4 [418.8 to 550.1]	41.1 [22.6 to 59.8]	**0.0001**

Blood samples were collected after an overnight fast and at least 12 hours after the last hydrocortisone dosage

Results are expressed as mean [95% CI] or median (interquartile range). Analyses are performed using paired *t*-tests. *P*-values < 0.05 are presented in bold

*PINP* serum type I N-terminal procollagen, *CTX* collagen type I cross-linked C-telopeptide, *OPG* osteoprotegerin, *RANKL* receptor activator kappa-B ligand

### Relation to quality of life

The primary outcome of this trial was previously published and showed that 9 of the 27 participants had improvements in fatigue above the scale-specific minimal importance changes [22]. In these nine participants, HbA_1c_ was lower after 16 weeks of DR-HC compared with C-HC (−1.6 mmol/mol [−3.0 to −0.2], *P* = 0.03). No difference was observed in salivary cortisone/cortisol, bone metabolism, body composition or cardiovascular outcomes in this group (data not shown).

## Discussion

In this prospective investigator-initiated switch trial, participants with secondary adrenal insufficiency were examined at baseline on stable C-HC and 16 weeks after a switch to DR-HC. After 16 weeks of DR-HC, late-evening salivary cortisol and cortisone decreased, and we found some evidence of lower diurnal salivary cortisol. Also, HbA_1c_ and total and abdominal fat mass decreased. These results confirmed previous findings that treatment with DR-HC seems to improve the metabolic profile of the participants compared with C-HC. However, we found no improvement in lipids, fasting blood glucose, and blood pressure, and the association between quality of life and the measured health variables was unclear.

Both supraphysiological replacement doses and the unphysiological timing of the replacement treatment seem to increase morbidity and mortality in patients with adrenal insufficiency [7, 31, 32].

The participants in this study received equivalent doses of C-HC and DR-HC [13], and the dose of DR-HC was titrated based on symptoms. Hence, the improvements in body composition and HbA_1c_ observed in the present and other studies may be a consequence of 24-hour cortisol concentrations and timing closer to normal when treated with DR-HC compared with C-HC treatment.

Still, the available oral hydrocortisone formulations cannot imitate the pulsatile rhythm of cortisol which seems to be of importance in regulating genes and normal cognitive function [33–35], but studies of patients with adrenal insufficiency are needed to further investigate these findings.

The diurnal cortisol exposure was evaluated by salivary cortisol and cortisone day curves and showed that the late-evening concentrations were lower after 16 weeks of DR-HC compared with baseline on C-HC. This is in line with other findings of both saliva [36] and serum [16]. Lower late-evening cortisol exposure could lead to an improved metabolic profile as elevated evening serum cortisol has been linked to the opposite [37]. Also, the present study found some evidence of a lower AUC of salivary cortisol as previously reported [36].

Saliva sampling is an easy and painless method to assess the diurnal cortisol concentrations and should, theoretically, reflect only free cortisol levels [38]. Both salivary cortisol and cortisone have been shown to reflect serum cortisol levels [39–41]. Still, the use of salivary cortisol/cortisone as a biomarker for monitoring glucocorticoid replacement treatment is debated [36, 39, 42].

Previous studies have shown a significant decrease in body weight and/or BMI [14–16, 19–21, 23, 25] which we could not confirm, but after 16 weeks of DR-HC, total and abdominal fat mass decreased.

Two studies [21, 25] investigated body composition assessed by bioelectrical impedance analysis but did not find any significant difference after the shift in treatment to DR-HC besides a positive correlation between the reduction in body weight and fat mass [21].

We showed a significant reduction in HbA_1c_ whereas no difference in fasting glucose and lipids was observed before and after 16 weeks of DR-HC treatment. Some previous studies [15–17, 20, 25, 26], but not all [18, 21, 23] observed a decrease in HbA_1c_. One study [18] showed a decrease in fasting glucose in patients with secondary adrenal insufficiency while others did not find a significant improvement [16, 17, 23, 25, 26]. Furthermore, fasting insulin and the Homeostatic Model Assessment for Insulin Resistance (HOMA-IR) decreased in one study [19] while others showed no difference after treatment [16, 23, 25, 26]. In this study, participants with diabetes were excluded to limit the number of confounding comorbidities. Yet, a previous study found that the decrease in HbA_1c_ was greater in participants with diabetes compared with participants without [16].

The glucometabolic improvements observed in the present and other studies may be a consequence of 24-hour cortisol timing closer to normal compared with C-HC treatment. This is in accordance with a study of healthy male participants in which the administration of 50 mg C-HC at 1700 h was associated with more pronounced hyperglycaemia and insulin resistance compared with the administration at 0500 h [37].

Our data showed no difference in lipids. Previous trials have found both improvement [15, 17, 20] and impairment [16, 21, 23, 25] of the lipid profile. Thus, the effect of DR-HC on the lipid profile remains unclear.

Previous studies have shown a reduction in blood pressure after treatment with DR-HC compared with C-HC [16, 20, 21] which this and other studies [17, 23] could not confirm, but, as the blood pressure did numerically decrease, our non-significant findings could be due to type II error. This study did, however, observe a reduction in blood pressure in the group of participants with hypertension.

We measured 24-hour and not office blood pressure which has been demonstrated to be a better predictor of cardiovascular risk [43] and which to our knowledge has not been investigated previously in patients treated with DR-HC.

In contrast with previous findings, the bone formation marker osteocalcin decreased after 16 weeks of DR-HC. The concentrations of PINP, CTX, OPG, and RANKL were unchanged. One prospective study [16] found an increase in the bone formation markers PINP and osteocalcin after 12 weeks of DR-HC treatment compared with C-HC, but patients were generally on higher doses of C-HC than in the present study. Also, two studies retrospectively observed an increase in BMD [18, 24] and the bone formation markers osteocalcin and bone alkaline phosphatase [24] after 12 and 60 months of DR-HC treatment compared with C-HC, respectively.

This study observed an increase in the Wnt signalling-inhibitor sclerostin after treatment with DR-HC compared with C-HC. The sclerostin concentration correlated positively with the replacement dose per kg body weight. No previous study has investigated sclerostin concentrations after treatment with DR-HC, but in endogenous hypercortisolism, studies have found an inverse association between sclerostin levels and the level of cortisol excess and suggested that the findings were caused by an inhibition of glucocorticoids on osteocyte function and/or number [44, 45]. Thus, our findings could indicate a decreased glucocorticoid inhibition on osteocytes after DR-HC compared to C-HC.

Both under- and overexposure to glucocorticoids impair bone health [46, 47]. It is unlikely that the participants were overtreated as the use of supplemental C-HC did not change in the study period and no relationship was found between the supplemental dose and bone status indices. It is also unlikely that the participants were undertreated as published data from this trial showed that fatigue was significantly reduced after 16 weeks of DR-HC which also did not indicate that the participants were undertreated [22].

Alternatively, the results were affected by other variables. Osteocalcin is affected by vitamin D and K, seasonal variation, and kidney function [48, 49]. Sclerostin varies with seasons [50], is inhibited by oestrogen, parathyroid hormone (PTH), and skeletal loading, and is stimulated by glucocorticoids, and skeletal unloading [51]. It is unlikely that oestrogen affected our results as only three postmenopausal women not substituted for oestrogen entered the study, but perhaps the reduction in fat mass and thus secretion of oestrogen could have increased sclerostin levels.

The present study is limited by a small sample size and consists of secondary outcomes from a previously published trial [22]. Also, only effects observed within the treatment time of 16 weeks could be evaluated in the present study. Furthermore, the trial was neither blinded nor randomised. Thus, the placebo effect could have affected the behaviour of the participants and their subjective responses i.e. to questionnaires and consequently have influenced the results as previously discussed [22].

Our study also had strengths. In a prospective study design, we examined a homogenous cohort of non-diabetic and primarily male participants diagnosed with secondary adrenal insufficiency treated with C-HC. Furthermore, the present trial included measurements of body composition by DXA, bone status indices, and 24-hour blood pressure that to our knowledge have not been published before.

As this and previous trials have not been placebo-controlled, the effect of DR-HC versus C-HC needs to be validated in a randomised, double-blind, placebo-controlled trial and perhaps compared with other glucocorticoids that have been proposed to mimic the circadian rhythm of cortisol.

In conclusion, this study confirms previous findings that switching from C-HC to DR-HC leads to lower late-evening cortisol and cortisone exposure and a more favourable glucometabolic profile and body composition in participants with secondary adrenal insufficiency. Discrepant results were found for bone status indices, the explanation for which remains unclear.

## Data Availability

No datasets were generated or analysed during the current study.
